# Laryngeal dystonia: a comprehensive review of aetiology, clinical presentation, diagnosis, and treatment approaches

**DOI:** 10.1007/s00405-026-10448-w

**Published:** 2026-07-16

**Authors:** Marius Tudor Ionescu, Maria Dana Morariu, Lucia Popa, Valeria Ionescu, Cristina Țiple, Septimiu Sever Pop, Daniela Vrînceanu, Alma Aurelia Maniu

**Affiliations:** 1 Department of Otorhinolaryngology, Iuliu Hațieganu University of Medicine and Pharmacy, Cluj-Napoca, Romania; 2 Vox Humana Centre for ENT and Phoniatrics, Enayati Medical City, Bucharest, Romania; 3 Department of Otorhinolaryngology, Bucharest University Emergency Hospital, Bucharest, Romania; 4 Department of Otorhinolaryngology, Colțea Clinical Hospital, Bucharest, Romania; 5 Department of Otorhinolaryngology, Cluj-Napoca County Emergency Hospital, Cluj-Napoca, Romania; 6 Department of Otorhinolaryngology, Carol Davila University of Medicine and Pharmacy, Bucharest, Romania

**Keywords:** Laryngeal dystonia, Spasmodic dysphonia, Laryngeal electromyography, Botulinum toxin

## Abstract

**Purpose:**

Laryngeal dystonia (LD), also known as spasmodic dysphonia, is a rare voice production pathology manifested as focal muscle dystonia. The aim of this review is to synthesize current evidence on its aetiology, clinical presentation, diagnostic approach, and available treatment options, while identifying existing knowledge gaps to guide clinical decision-making and future research.

**Methods:**

A structured literature review was conducted using peer-reviewed articles from medical databases (e.g., PubMed, Google Scholars). Inclusion criteria comprised studies published in English within the last 20 years, focusing on LD aetiology, clinical features, diagnosis, or treatment. Both observational studies and clinical trials were included. Exclusion criteria involved case reports with limited applicability, non-English publications, and studies lacking clear diagnostic criteria or therapeutic outcomes.

**Results:**

Primarily an idiopathic disorder, there is evidence suggesting a neurogenic origin involving basal ganglia dysfunction in LD. Clinically, LD manifests as task-specific voice disturbances, affecting most commonly the adductor muscles (strained voice) or the abductor muscles (breathy voice). Diagnosis is mainly clinical, but supported by laryngoscopic findings and other multidisciplinary evaluation. The gold standard treatment remains botulinum toxin injection, providing symptomatic relief, while voice therapy and, in selected cases, surgical interventions offer adjunctive benefits.

**Conclusion:**

LD is a neurological voice disorder which requires a high index of suspicion for diagnosis. Individualized treatment, particularly with botulinum toxin, and early recognition, significantly improve patient outcomes. Further research is needed to clarify pathophysiological mechanisms and optimize long-term management strategies.

## Introduction

Dystonias are defined as movement disorders characterized by involuntary muscle contractions causing uncontrollable spasms during an activity [1]. The most frequently encountered form of this disorder is focal dystonia, in which the muscle groups in a single body region are affected. One such type of focal dystonia is the laryngeal form, also referred to as spasmodic dysphonia, characterized by spasm of laryngeal musculature during speaking. Depending on which muscles it affects, laryngeal dystonia (LD) may be classified as adductor (ADLD), abductor (ABLD), and mixed type [2].

In the adductor type, excessive closing of the vocal folds during phonation produces a strained-strangled voice quality with vocal breaks, especially on words beginning with vowels or on vowels in the middle of a word. ADLD is the most prevalent type, affecting approximately 80% of LD patients [3]. The much rarer abductor type manifests as uncontrolled spasms in the muscles that help open the vocal folds, producing a breathy voice quality occurring predominantly on voiceless consonants. Mixed LD combines features of both adductor and abductor types.

First reported in 2006 by Chitkara and colleagues [4], singer’s laryngeal dystonia (SLD) is a rare subtype of focal laryngeal dystonia presenting with features characteristic of either ADLD or ABLD during specific tasks in the professional singing voice. Another subtype of ADLD described in the literature is the adductor respiratory laryngeal dystonia. This is the less frequent type and also the more concerning one, since it presents with persistent respiratory stridor and cough, usually without dysphonia during speech [5].

## Aetiology

### Historical psychogenic theory

LD was historically mischaracterized as a “psychogenic” or “functional” voice disorder. In the early and mid-twentieth century, patients with LD were often thought to suffer from a form of hysterical dysphonia or conversion disorder, given the task-specific nature of their symptoms and intermittent normal voice in contexts like laughing or singing. However, a characteristic feature of laryngeal dystonia is that the spasms are typically not present in innate vocal production, such as laughing, crying, whispering or yawning. [6]. This paradigm shift laid the groundwork for current research into the organic causes and mechanisms of LD.

### Genetic contributors

A subset of patients shows familiar aggregation of dystonia or essential tremor, indicating an inherited predisposition, although true familiar LD is rare. Several gene mutations associated with primary dystonias have been linked to LD phenotypes. Notably, mutations in the THAP1 gene (DYT6 dystonia) have been identified in families where LD co-occurs with other dystonic features, and a distinct mutation in the TUBB4A gene (DYT4 “whispering dysphonia”) was discovered in a family presenting with a hereditary form of laryngeal dystonia [7]. These findings suggest that in rare cases LD can be one manifestation of a broader genetic dystonia syndrome. Overall, no single gene is known to cause most cases of LD, but ongoing genetic studies indicate that LD likely shares polygenic risk factors common to focal dystonias.

### Environmental contributors

Environmental and behavioural factors are thought to act as triggers in individuals who are genetically susceptible. Many patients date the onset of LD symptoms to a specific precipitating event or exposure. Upper respiratory tract infections have been frequently reported preceding LD onset – one survey found roughly 30% of patients attributed their symptom onset to a recent cold or viral illness. Likewise, significant psychological stress or trauma around the time of onset was reported by about 20% of patients [6], which, while not a primary cause, may exacerbate or unmask an underlying neurological predisposition. Occupational voice strain has also been implicated: case–control studies observed that intensive voice use (for example, among teachers or singers) is significantly more common in LD patients, suggesting that heavy and prolonged vocal demand might trigger dystonic symptoms in a vulnerable laryngeal motor system. Other exposures, such as specific infections (e.g. mumps) or even neck injuries, have been noted as potential risk factors in some studies [8]. It is important to emphasize that these environmental factors are common in the general population and not sufficient by themselves to cause LD; rather, they likely interact with genetic predispositions in a multifactorial pathogenic process.

### Neuroimaging findings

Neuroimaging research has provided strong evidence that LD reflects a disorder of central neural networks for voice control. Structural MRI and diffusion tensor imaging studies have detected subtle neuroanatomical differences in the brains of LD patients. For instance, localized white matter changes have been demonstrated along the corticobulbar pathways subserving laryngeal movement, as well as in key sensorimotor regions and basal ganglia structures involved in voice production [9]. Functional neuroimaging (fMRI) and PET investigations have documented abnormal functional connectivity and metabolism in the laryngeal sensorimotor cortex, supplementary motor area, and cerebellar networks, supporting the concept that LD patients have abnormal activation patterns in cortical and subcortical voice areas, involving a large-scale brain network disorganization [10]. These neuroimaging findings collectively suggest that LD is rooted in dysfunction of the central speech motor network, involving impaired communication between the cortical centres for speech planning/execution, the basal ganglia inhibitory circuits, and possibly the cerebellar modulatory pathways.

### Neurophysiological abnormalities

Neurophysiological studies of LD corroborate the imaging evidence of a network-level motor control disorder. In particular, LD exhibits the hallmark electrophysiological abnormalities seen in other dystonias. Transcranial magnetic stimulation (TMS) studies have shown a loss of normal intracortical inhibition in LD patients [11]. This mirrors findings in other focal dystonias (e.g. hand and cervical dystonia) and suggests that deficient inhibition contributes to the pathogenesis, allowing excessive or unopposed motor excitation during speech. There is also evidence for aberrant sensorimotor integration in LD. Patients demonstrate impaired sensory processing of laryngeal feedback; for example, studies have noted abnormal responses to somatosensory stimulation of the larynx and altered auditory feedback processing during voice tasks in LD. Overall, the neurophysiological profile of LD – characterized by reduced cortical inhibition and abnormal sensorimotor integration – reinforces its status as a neurogenic movement disorder and provides objective biomarkers that distinguish LD from psychogenic voice disorders [11].

## Diagnosis and clinical presentation

Laryngeal dystonia develops without warning or any clear preceding event with the average onset in the range of 39–45 years of age. Some patients report a gradual onset of symptoms, whereas others describe a sudden onset, often associated with a stressful event or an upper respiratory infection. Around 18% of patients present with dystonia to other body regions, while the remaining 82% have a laryngeal only presentation [12].

Even for the most experienced laryngologists, diagnosing LD might prove difficult as there is no diagnostic criteria or scientific consensus. The main diagnostic challenge in laryngeal dystonia lies in distinguishing it from other voice disorders with overlapping clinical presentations, such as vocal tremor and muscle tension dysphonia (MTD). This differentiation is critical, as treatment strategies differ significantly: MTD typically responds well to voice therapy, whereas LD does not [13]. To address this diagnostic challenge, Ludlow and colleagues [14] identified the need for a standardized diagnostic approach using a three-tiered system: 1) screening questions to reveal *possible* LD, 2) speech examination to recognise *probable* LD and 3) videostroboscopy for *definite* diagnosis and excluding other laryngeal pathologies.

An affirmative answer to the first two screening questions (Table 1, adapted from Ludlow et al. [14]) and a duration of symptoms of at least 3 months are required for a possible LD diagnosis. Patients should then be referred to examination by a specialist, who will perform a series of eliciting speech exercises to further identify the probability of LD. For a positive diagnosis, more than 1 voice break per 3 sentences in a normal speaking voice and none in a whispered voice are required. The last step of Ludlow’s three-tiered approach includes laryngeal examination by videostroboscopy to exclude other causes for the voice symptoms and for definite diagnosis. The required visual features for diagnosis include: no anatomical defects to account for the abnormal voice and a normal motion of the vocal folds during vegetative laryngeal tasks such as coughing, throat clearing, yawning and whistling. Using this three-tiered system, Ludlow and colleagues were able to correctly categorize 97% of patients with known LD, vocal tremor or MTD, from a group of 30 patients [14].

**Table 1:** Screening questions for laryngeal dystonia (LD)

Question	Typical Response for LD	Response Unlikely for LD
1. Do you find speaking effortful or tiring?	Yes	No
2. Does your speaking ability fluctuate (easier at times, harder at others)?	Yes	Occasionally normal without treatment
3. How long have you experienced difficulty speaking?	3 months or longer (chronic)	Less than 3 months
4. Can you perform the following tasks normally?		
- Shouting	Yes	No
- Crying	Yes	No
- Laughing	Yes	No
- Whispering	Yes	Similar difficulty as normal speech
- Singing	Yes	Worse than normal speech
- Yawning	Yes	No

Source adapted from: Ludlow CL, Adler CH, Berke GS, Bielamowicz SA, Blitzer A, Bressman SB, et al. Research priorities in spasmodic dysphonia. Otolaryngol–head neck surg. 2008 Oct;139(4):495–505

In the diagnosis of difficult cases, objective testing and additional diagnostic modalities can serve as valuable components in the armamentarium of the laryngologist. One such additional tool is laryngeal electromyography (LEMG). This procedure involves inserting fine needles into the intrinsic laryngeal muscles – such as the thyroarytenoid, posterior cricoarytenoid, and cricothyroid muscles – to record electrical activity at rest and during various phonatory and non-phonatory tasks, allowing clinicians to assess muscle function under dynamic conditions.

The diagnostic utility of LEMG lies in its ability to provide objective evidence of neuromuscular dysfunction. In cases of LD, the examination often reveals abnormal recruitment patterns such as large polyphasic motor unit potentials, prolonged latencies between the onset of electrical activity and sound production and inappropriate bursts of activity during speech tasks [15]. Since none of these findings are pathognomonic for LD, LEMG results must be interpreted in the context of the clinical picture.

Several studies have significantly advanced our understanding of dystonic patterns within the larynx. For example, Klotz and co-workers [16] demonstrated in a cohort of 214 patients that multiple intrinsic laryngeal muscles are frequently simultaneously affected, indicating more complexity in the disease than previously assumed. Supporting these observations, Cyrus et al. [17] reported paradoxical activation of the thyroarytenoid muscle even in abductor LD (ABLD), where diminished adductor activity is typically expected. More recently, Yang et al. [18] concluded that LEMG is a valuable tool as a diagnostic adjunct of LD and can also objectively evaluate treatment efficacy. These findings collectively underscore that a comprehensive LEMG evaluation can reveal unexpected and heterogeneous muscle involvement, which is essential for tailoring therapeutic interventions such as targeted botulinum toxin injections.

Complementing LEMG, basic phonatory measures such as maximum phonation time (MPT) – the longest duration a patient can sustain a vowel sound – have also been useful in diagnosing LD. Since the vocal folds involuntarily over-adduct in ADLD patients, they may exhibit a longer MPT compared to normal voices (due to reduced air leakage), whereas ABLD patients may show a reduced MPT due to excessive airflow escape caused by the over-abduction of the vocal folds. Illustrating this, Nerurkar and Goyal [19] found prolonged (> 24 s) MPT values in ADLD patients and reduced (< 10 s) MPT values in ABLD patients, compared to healthy controls (16 s). These findings led the authors to recommend including MPT as a low-cost, objective measure in the diagnostic workup of LD.

Acoustic analysis is another integral objective method for characterising the status of vocal function. Classical perturbation measures such as jitter (frequency), shimmer (amplitude), and harmonics-to-noise ratio (HNR) can be employed to characterise voice dysfunction in LD, but often lack specificity as they overlap with other dysphonic disorders [20]. This limitation has motivated the search for more robust acoustic markers and composite indices that integrate multiple acoustic parameters.

One promising composite metric is the Acoustic Voice Quality Index (AVQI), which combines analyses of connected speech and sustained vowels to measure the overall quality of the voice. In laryngeal dystonia, AVQI has shown promise as an objective marker for voice impairment: for example, a comparative study found that AVQI scores differentiated LD patients from normophonic individuals with statistically significant separation (*p* < 0.05) [21].

Further advancements have been achieved with cepstral analysis, a sophisticated frequency-domain technique for quantifying voice quality and periodicity. Suppa et al. [22] successfully employed cepstral-spectral features and machine learning algorithms to differentiate LD voices from controls, highlighting the diagnostic potential of advanced acoustic analyses.

Contemporary research is exploring novel acoustic metrics to improve the detection and monitoring of LD. One recent innovation is the global dimension (GD) acoustic metric, introduced by Liu et al. [23] as an objective indicator for ADLD. This method is based on nonlinear dynamics analysis of the voice signal, demonstrating superior performance in distinguishing ADLD from normal voices compared to conventional measures such as jitter and shimmer, achieving excellent diagnostic accuracy.

Collectively, these advanced acoustic tools hold great potential for objectively quantifying the presence, severity and clinical progression of laryngeal dystonia. However, the typical clinical diagnosis of LD continues to rely on recognizing the characteristic voice features and excluding other pathologies. Integrating objective diagnostic modalities such as LEMG, acoustic analysis, genetic testing and advanced imaging techniques serves at enhancing our understanding and management of this complex condition.

## Current treatment approaches

To date, no permanent cure for LD exists, but several treatment approaches are available to alleviate or manage the symptoms. Interest in laryngeal dystonia as a medical condition significantly advanced with Dedo’s [24] introduction of unilateral recurrent laryngeal nerve (RLN) section as a surgical intervention, based on initial success with temporary lidocaine nerve block in 1976. Although widely adopted, this procedure often resulted in symptom recurrence attributed to reinnervation of the recurrent nerve [25]. Subsequent surgical techniques, such as myectomies, unilateral RLN avulsion, bilateral denervation with reinnervation of the thyroarytenoid muscles, and various laryngoplasty procedures, emerged as attempts to achieve more durable outcomes [26]. However, results remained inconsistent, with side effects including voice roughness, persistent breathiness and unpredictable long-term efficacy.

Following its successful use in treating blepharospasm and torticollis, two focal dystonias affecting the periocular and cervical muscles, botulinum toxin (BoNT) was introduced as a treatment for laryngeal dystonia in 1984. Blitzer’s prior experience with laryngeal electromyography enabled precise delivery of the toxin into the intrinsic laryngeal muscles, effectively performing a chemical neurectomy that alleviated symptoms. This treatment relies on the same principle of denervation as recurrent nerve section, but offers the advantage of temporary, yet repeatable symptomatic relief [27].

### Botulinum toxin (BoNT) injection

Chemodenervation with BoNT injection is currently the gold-standard therapy for laryngeal dystonia and has been the primary treatment method for over three decades [28]. BoNT (usually type A) is injected percutaneously into the overactive intrinsic laryngeal muscles to cause a local, temporary paresis. The mechanism is a reversible blockade of acetylcholine release at the neuromuscular junction, inducing flaccid paralysis of the targeted muscle [29]. This controlled weakening of hyperactive laryngeal muscles relieves the strained, intermittent contractions that underlie LD, thereby improving voice fluency.

Despite its efficacy, BoNT therapy is not curative and has practical limitations. The effect is temporary, lasting on average 3–4 months and requires periodic reinjections to maintain voice improvement. Patients often experience a short period of side-effects after each injection, most commonly transient breathy voice and mild dysphagia for liquids due to diffuse weakening of the laryngeal muscles [30]. These side effects typically self-resolve within a few weeks and are often regarded as an expected part of the treatment cycle. Occasionally, patients may develop partial tolerance over time, possibly from neutralizing antibody formation, leading to shorter duration or reduced benefit.

Over the years, clinicians have developed several injection techniques to improve accuracy and patient comfort. The traditional technique uses laryngeal electromyography guidance to confirm correct needle placement before the toxin is injected [29]. This approach can achieve excellent results, but it requires specialized equipment and expertise in interpreting EMG signals.

An alternative to EMG guidance is the transnasal endoscopic approach. In this method, a flexible nasopharyngoscope is passed through the patient’s nose to visualize the larynx from above. A very thin needle is then used to inject the target muscle under direct visualization, either through the working channel of the endoscope or percutaneously. A recent retrospective report including 8 ADLD patients (20 total injections) showed that office-based transnasal endoscopic BoNT injection is feasible, safe and effective [31].

The point-touch technique relies on tactile anatomical landmarks on the larynx to guide needle placement rather than electrical or visual feedback. It involves palpating external landmarks and inserting the needle at a defined point and depth to reach the TA muscle. In a prospective series of ADLD patients [32], point-touch BoNT injections led to significant voice quality improvements with only mild, transient dysphagia post-injection. Patient-reported voice-related quality of life improved significantly, which led the authors to conclude that this method is a viable alternative to EMG-guided injections.

Finally, ultrasound (US) imaging is emerging as a useful tool to visualize laryngeal structures in real-time during BoNT injection. This has been described especially for ABLD, where the target posterior cricoarytenoid (PCA) muscle is located deep in the neck. A recent case report [33] demonstrated successful BoNT injection into the PCA under transcutaneous cervical US guidance. The sonography can improve injection accuracy by visualizing the needle-path in real time and ensuring the toxin is not injected into unintended structures. Beyond case reports, an experimental pilot study in cadavers demonstrated that US-guided injections into the lateral cricoarytenoid (LCA) muscle, one of the adductors involved in ADLD, achieved accurate targeting in 90% of cases [34]. These findings support US as a reliable, non-invasive alternative to EMG for guiding laryngeal injections. While operator skill and anatomical challenges may limit its use, growing access to portable US may expand its application for both ADLD and ABLD.

While dysphagia and breathiness remain the most frequently reported side effects of BoNT injections for laryngeal dystonia, clinicians should also be aware of rarer but potentially serious complications. Airway compromise, though exceedingly uncommon, has been reported in the literature. One such case report involved a patient who developed acute respiratory distress and stridor approximately 10 days post-injection, ultimately requiring intubation [35]. The patient had received a routine dose, and the severe vocal fold adduction was believed to result from an idiosyncratic response, possibly due to rebound hypertonicity. Another recently recognised side effect is obstructive sleep apnea (OSA). There is emerging evidence suggesting that reduced laryngeal muscle tone following BoNT injection may contribute to upper airway collapse during sleep. A case report [36] described a patient with ADLD who exhibited new-onset OSA on polysomnography exam after treatment.

In scenarios where severe complications may occur, pyridostigmine, a reversible acetylcholinesterase inhibitor, may serve as a pharmacological antidote. Pyridostigmine enhances neuromuscular transmission by preventing the breakdown of acetylcholine, thereby counteracting the paralytic effect of BoNT. Therefore, it may be considered as a salvage therapeutic option in carefully selected patients experiencing profound muscle weakness secondary to BoNT administration [37].

BoNT into the ventricular (false) vocal folds has been investigated as an alternative approach for ADLD to address supraglottic hyperadduction and reduce the side effects of standard TA muscle injections. Early case reports demonstrated that weakening the false vocal folds (FVF) can dramatically improve a hyperadducted voice; for example, Rosen and Murry [38] reported a patient with severe FVF hyperadduction who achieved near-normal voice quality after bilateral FVF BoNT injections, with resolution of FVF closure during phonation. Building on such findings, Young and Blitzer [39] introduced a technique of injecting BoNT into the supraglottic portion of the LCA muscles (effectively targeting the false folds) for ADLD patients exhibiting a “supraglottic squeeze” (compensatory FVF contraction) that persisted despite TA injections. All four patients in their series showed improved voice quality compared to TA injection alone, and the procedure was performed safely in an office setting [39]. Subsequent larger studies have confirmed the rationale and efficacy of FVF injections. Simpson et al. (2016) reported that in a cohort of ADLD patients treated primarily with FVF BoNT, voice function significantly improved (mean post-injection approximately 95% of normal vs approximately 62% pre-injection, *p* < 0.001) and the majority (76%) did not experience the typical breathy voice weakness that follows TA injections. Patients’ Voice Handicap Index scores after treatment were in the normal range, underscoring the effective symptom control [3]. In Simpson’s series the duration of benefit per injection was on the order of 3–4 months (comparable to standard injection cycles). A more recent comparative study by Larsen et al. [40] analyzed patients who switched from TA to FVF injections. They found that FVF injections achieved similar voice outcomes to TA injections, with comparable duration of “best voice” and no increase in breathiness or dysphagia symptoms reported post-injection. However, the injection effect duration was somewhat shorter with FVF injections – the average injection cycle became significantly shorter than in those continuing TA injections, indicating the toxin’s effect wore off faster and reinjections were needed more frequently. A combined approach (injecting both the FVFs and a reduced dose into the TA) yielded voice quality and side-effect profiles equivalent to standard TA injection alone [40]. Safety appears favorable: the FVF injection technique has been well tolerated without serious adverse events in reports [39]. Overall, the literature supports FVF BoNT injection as a valid and safe treatment option for ADLD – it yields voice quality improvements comparable to the standard TA injection, with a distinct side-effect profile (less post-injection breathiness at the cost of possibly shorter action) that can be advantageous in appropriately selected patients.

Overall, BoNT remains a well-tolerated and highly effective treatment when performed by experienced clinicians. Most side effects are mild, temporary and manageable, however appropriate counselling and follow-up are essential to ensure patient safety and satisfaction.

### Surgical interventions

Given the transient nature of BoNT efficacy in laryngeal dystonia, several surgical procedures have been developed to achieve more durable symptom control. These interventions aim to denervate, weaken, or structurally alter the vocal fold muscles, paralleling the mechanism of BoNT-induced chemodenervation. Principal surgical strategies include recurrent laryngeal nerve (RLN) section, which induces vocal fold paralysis via nerve transection; thyroarytenoid (TA) myectomy [41] or myoneurectomy [42], which involves selective resection or ablation of the TA muscle to limit hyperactivity; type II thyroplasty [43], which mechanically lateralizes the vocal folds to counteract hyperadduction; and selective laryngeal adductor denervation-reinnervation (SLAD-R), in which targeted neural input to adductor muscles is interrupted and redirected to reduce aberrant reinnervation [44].

All of the aforementioned surgical interventions aim to improve phonatory fluency and reduce the strained, effortful voice characteristic of LD, though they differ in their underlying mechanisms of action. Denervation-based procedures, such as RLN section, SLAD-R and nerve crush techniques target the neural input to the laryngeal muscles. Among these, SLAD-R has demonstrated superior long-term efficacy by combining targeted denervation with functional reinnervation, thereby minimizing the risk of aberrant reinnervation [45].

Muscle-directed interventions, including thyroarytenoid myectomy, myoneurectomy, and radiofrequency myothermy, aim to reduce the muscle’s ability to spasm. These approaches have shown comparable efficacy to SLAD-R in various case series [42], although some patients may require repeat procedures to sustain therapeutic benefit. Laryngeal framework modifications – such as type II thyroplasty, and reconstructive strategies – such as autologous vocal fold replacement, alter glottic biomechanics. Type II thyroplasty has proven highly effective in appropriately selected patients by reducing glottic hyperadduction [43], while autologous replacement remains investigational but conceptually promising.

Transoral radiofrequency-induced thermotherapy (RFITT) is a minimally invasive technique targeting distal branches of the RLN innervating the TA muscle, functioning as a focal denervation. In a series by Beyaert et al. [46], 11 patients with ADLD showed significant voice improvement after a single session, measured by the Voice Handicap Index. The mechanism is similar to SLAD-R, but it achieves denervation through thermal lesions rather than nerve transection.

In terms of long-term outcomes, SLAD-R, type II thyroplasty, and laser-assisted myoneurectomy procedures report sustained symptomatic improvement in approximately 80–90% of patients over multi-year follow-up periods [45]. In contrast, isolated RLN section has demonstrated diminished efficacy over time due to spontaneous reinnervation and is now considered largely historical.

### Neuromodulation approaches

Both BoNT-A injections and laryngeal surgeries aim to alleviate the symptoms of laryngeal dystonia by targeting the laryngeal musculature. BoNT-A induces temporary chemodenervation of hyperactive adductor muscles, while surgical procedures aim for more permanent alteration. Although the precise pathophysiological mechanisms underlying LD remain incompletely understood, it is now well established that LD is a centrally mediated neurological disorder. Given this central aetiology, neuromodulation has emerged as a potential therapeutic strategy in which targeted modulation of neural activity via electrical or pharmacological stimuli is achieved. As such, this may offer durable symptomatic improvement beyond the peripheral effects of current treatments.

Vibro-tactile stimulation (VTS) involves applying vibratory stimuli to the skin overlying the larynx. The rationale is partly akin to the sensory trick (“geste antagoniste”) phenomenon observed in dystonia, where specific sensory input can transiently reduce dystonic muscle activity. In a recent 11-week trial, Konczak et al. [47] showed that both superficial (40 Hz) and deep (100 Hz) vibratory stimulation applied once or three times per week significantly improved voice quality and reduced vocal effort in patients with ADLD. VTS appears to desynchronize pathologically synchronized cortical activity in LD, contributing to improved motor output.

Peripheral electrical stimulation has also been proposed as a means of overriding aberrant laryngeal muscle activity. Pitman [48] investigated an implanted electrical stimulation device acting as a laryngeal pacemaker. In a pilot cohort of five patients with ADLD, three experienced global improvement in voice function, with one partial responder. Notably, the treatment produced benefits without the breathy voice commonly associated with BoNT therapy.

Repetitive transcranial magnetic stimulation (rTMS) applies pulsed magnetic fields to the cortex to modulate neuronal excitability. In dystonias, low-frequency stimulation (≤ 1 Hz) is typically used to enhance intracortical inhibition, which is often impaired. In a small pilot study, Prudente et al. [49] demonstrated the safety and feasibility of 1 Hz rTMS targeting the laryngeal motor cortex. Patients showed improvements in voice stability and reduction in phonatory breaks after a single session, with no adverse effects. The rTMS approach is hypothesized to restore deficient intracortical inhibition found in LD, but further controlled trials are warranted to establish its role in therapy [50].

Deep brain stimulation (DBS) is an established therapy for severe generalized or cervical dystonias, typically targeting the globus pallidus internus (GPi). While not standard for focal dystonias such as laryngeal dystonia, preliminary studies have explored its use, particularly targeting the thalamus. Early evidence includes a case series by Evidente et al. [51], in which three patients with ADLD showed notable voice improvement after bilateral DBS of the ventral intermediate nucleus (Vim) of the thalamus. A subsequent Phase I trial [52] implanted unilateral Vim-DBS in six patients, showing improved voice quality and quality of life during blinded assessments. Long-term follow-up suggested sustained benefit. Currently, DBS for LD remains investigational and further research is needed to refine targets and establish efficacy before routine clinical adoption.

Sodium oxybate (Xyrem®) is a medication rather than a device-based intervention, but it represents a pharmacological neuromodulator that has shown promise in LD. Sodium oxybate is a CNS depressant that is used in treating narcolepsy with cataplexy or excessive daytime sleepiness [53]. Chemically, sodium oxybate is the sodium salt of gamma-hydroxybutyrate (GHB) and a GABA-B receptor agonist. Interest in this medication for treatment of LD arose from the observation that over half of LD patients report transient voice improvement with alcohol – an effect it may replicate due to overlapping GABAergic mechanisms [54].

In a 2013 case report, Simonyan and Frucht [55] described a patient with LD and concurrent vocal tremor who experienced near-complete symptom remission lasting 10 months following a single dose of sodium oxybate. During this prolonged remission, functional imaging showed normalized activity in key regions of the brain’s dystonia network, suggesting a profound neuromodulatory impact from the drug [55].

To systematically assess efficacy, Rumbach et al. [56] conducted an open-label trial in which they showed that a single dose of sodium oxybate produced rapid (30–40 min onset) and significant improvement in voice quality in over 80% of alcohol-responsive LD patients, with effects lasting approximately four hours.

While its short duration and sedative profile limit daily use, sodium oxybate may be useful as an on-demand treatment (e.g. before public speaking). Nonetheless, its efficacy highlights the GABAergic system as a viable target in LD management.

## Conclusion

Laryngeal dystonia (LD) is an isolated focal dystonia of the laryngeal muscles, characterized by task-specific involuntary spasms during speech that cause intermittent voice breaks and strained or breathy voice quality. Two main subtypes are recognized: the more common adductor type, which produces a strained, strangled voice with intermittent breaks due to involuntary vocal fold closure, and the less common abductor type, which causes periodic breathy breaks due to involuntary vocal fold opening. Mixed or intermediate presentations and concomitant vocal tremor also occur in some patients, reflecting a heterogeneous clinical spectrum.

The aetiology of laryngeal dystonia is multifactorial and neurogenic. Dysfunction in central motor control networks (including basal ganglia and cortical circuits) is thought to underlie the loss of normal voice control. A genetic component is suggested by occasional familial cases and rare identified mutations, although most cases are sporadic. Environmental or psychosocial triggers (such as stress or viral illness) are often reported around symptom onset, but likely only precipitate symptoms in a predisposed individual.

Diagnosing LD is challenging because its symptoms overlap with other voice disorders and there is no definitive test. It requires expert clinical evaluation, often with fiberoptic laryngoscopy during speech to visualize the characteristic spasms that coincide with voice breaks. Typically, these spasms occur during speech while non-speech vocalizations (laughing, coughing, whispering) remain unaffected, a pattern that helps distinguish LD from functional dysphonia. In uncertain cases, laryngeal electromyography or acoustic analysis may provide supporting information, but diagnosis ultimately relies on clinical judgment.

Treatment of LD focuses on symptomatic management aimed at improving voice function. Botulinum toxin injections into the overactive laryngeal muscles are the gold standard, producing significant voice improvement for most patients. The effect is temporary (lasting a few months), so repeated injections are needed to maintain benefit. For severe or unresponsive cases, surgical interventions such as selective laryngeal nerve denervation-reinnervation or myoneurectomy have been employed to provide longer-lasting relief by permanently disrupting the dystonic signals to the vocal folds, though these procedures are reserved for carefully selected patients and outcomes can vary.

Emerging therapies such as neuromodulation are also being explored. Preliminary studies of deep brain stimulation and noninvasive brain stimulation suggest these techniques may help modulate disordered voice control in severe cases, but they remain experimental. At the same time, supportive care is important: voice therapy can help patients optimize their vocal technique and cope with symptoms. Some individuals also pursue complementary approaches like acupuncture or relaxation techniques; these may offer subjective benefit, but evidence for their efficacy is limited.

In conclusion, effective management of laryngeal dystonia requires an individualized, multidisciplinary approach integrating medical, surgical, and rehabilitative strategies. While current treatments—especially botulinum toxin injections—offer significant symptomatic relief, no definitive cure exists, underscoring the need for further research into the disorder’s pathophysiology. Future advances in understanding the neural mechanisms and genetic factors of laryngeal dystonia are critical to developing targeted, disease-modifying therapies. Ongoing efforts to refine diagnostic tools and improve therapeutic interventions, combined with interdisciplinary collaboration, will be essential for enhancing long-term outcomes for patients with this challenging voice disorder.

## Declarations

### Data Availability

No new datasets were generated or analysed during the current study. Data sharing is not applicable to this article.
